# A Point-Scoring System for the Clinical Diagnosis of Sjögren's Syndrome Based on Quantified SPECT Imaging of Salivary Gland

**DOI:** 10.1371/journal.pone.0155666

**Published:** 2016-05-19

**Authors:** Jing Chen, Xia Zhao, Haixia Liu, Sheng Zhou, Yunqiang Yang, Shouxin Li, Zhiqun Xianyu, Yunfeng Han, Guifen Shen, Jinming Li, Cong Ye, Wei Sun, Lingli Dong

**Affiliations:** 1 Department of Nuclear Medicine, Tongji Hospital, Tongji Medical College, Huazhong University of Science and Technology, Hubei, Wuhan, China; 2 Institute of Pathology, Tongji Hospital, Tongji Medical College, Huazhong University of Science and Technology, Hubei, Wuhan, China; 3 Department of Ophthalmology, Tongji Hospital, Tongji Medical College, Huazhong University of Science and Technology, Hubei, Wuhan, China; 4 Department of Stomatology, Tongji Hospital, Tongji Medical College, Huazhong University of Science and Technology, Hubei, Wuhan, China; 5 Department of Rheumatology and Immunology, Tongji Hospital, Tongji Medical College, Huazhong University of Science and Technology, Hubei, Wuhan, China; 6 Department of Stomatology, Union Hospital, Tongji Medical College, Huazhong University of Science and Technology, Hubei, Wuhan, China; Penn State University, UNITED STATES

## Abstract

**Objective:**

To establish a point-scoring diagnostic system for Sjögren's syndrome (SS) based on quantified SPECT imaging of salivary gland, and evaluate its feasibility and performance compared with 2002 AECG criteria and 2012 ACR criteria.

**Methods:**

213 patients with suspected SS enrolled in this study. The related clinical data of all patients were collected. All patients were evaluated and grouped on a clinical basis and posttreatment follow-up by rheumatology specialists as the unified standard (SS group with 149 cases and nSS group with 64 cases). From SPECT imaging of salivary gland, T_max_, UI_max_, T_s_ and EF_s_ were derived for bilateral parotid and submandibular glands, and compared between the groups. A point-scoring diagnostic system for SS was established based on the quantified SPECT imaging of salivary gland. We estimated the sensitivity, specificity, positive predictive value (PPV), negative predictive value (NPV) and accuracy for the new diagnostic system, compared with 2002 AECG criteria and 2012 ACR criteria.

**Results:**

When 7.0 was used as the cut-off point, the sensitivity, specificity, PPV, NPV and accuracy for the new point-scoring system in diagnosing SS were 89.93% (134/149), 93.75% (60/64), 97.10% (134/138), 80.00% (60/75) and 91.08% (194/213), respectively. The new point-scoring diagnostic system based on quantified SPECT imaging of salivary gland keeps the specificity comparatively to 2002 AECG criteria and 2012 ACR criteria, but improves the sensitivity significantly (*P*<0.01).

**Conclusion:**

The new point-scoring diagnostic system for SS based on quantified SPECT imaging of salivary gland may be superior to 2002 AECG criteria and 2012 ACR criteria, with higher sensitivity and similar specificity in the diagnosis of SS. Additionally, it also has good feasibility in the clinical settings.

## Introduction

Sjögren's syndrome (SS) is a systemic progressive autoimmune disease with external exocrine glands dysfunction and multiorgan involvement. The salivary and lacrimal glands are the most affected glands [1]. Despite being the second most common autoimmune disease and overlapping in 15–30% of patients with other autoimmune disease, the diagnosis of SS may be difficult to establish because there is no gold standard test [2,3].

For research purposes, several sets of classification criteria have been proposed [4,5,6,7]. The classification criteria for SS issued in 2002 revised by the American-European Consensus Group (2002 AECG criteria) is the most widely used criteria in clinical studies over the last decade [2]. And another classification criteria for SS proposed in 2012 by the American College of Rheumatology (2012 ACR criteria) have recently been endorsed [8]. However, all published classification criteria for SS, including 2002 AECG criteria and 2002 ACR criteria, have a high specificity but low sensitivity [4,9,10]. In addition, agreement was only moderate between 2002 AECG criteria and 2012 ACR criteria [11,12]. And 38% of patients with a clinical diagnosis of SS were missing by 2002 AECG criteria or 2012 ACR criteria. Even if both criteria are applied in parallel, 20% of SS patients were still missing [5].

The diagnosis criteria based on point-scoring system can improve the sensitivity and specificity in different diseases, such as 2010 rheumatoid arthritis classification criteria [13] and systemic sclerosis classification criteria [14]. The score in the point-scoring system reflects the weight of each criterion which parallels its specificity. Our current study aims to establish new point-scoring diagnostic system for SS based on the quantified SPECT imaging of salivary gland (SSG), and evaluate its feasibility and performance compared with 2002 AECG criteria and 2012 ACR criteria.

## Materials and Methods

213 patients (192 women, 21 men; age range, 13–84 y; mean age, 46.16 y) with suspected SS consulting in the department of Rheumatology and Immunology at Tongji Hospital of Tongji Medical College, Huazhong University of Science & Technology (HUST) between January 1^st^, 2012 and April 30^th^, 2015 were enrolled in this study. The following data were collected including previous medical history, subjective complaints of oral and ocular dryness, ocular examination(ocular staining score or Schirmer’s I test), quantitative parameters from SSG, serological tests including anti-SSA/Ro, anti-SSB/La, antinuclear antibody (ANA) and rheumatoid factor (RF), and minor salivary gland biopsy. All patients were evaluated and divided into two groups on a clinical basis and posttreatment follow-up by three rheumatology specialists: group A (SS) and group B (nSS).

The study was approved by the local ethics committee at Tongji Hospital of Tongji Medical College, HUST (Permit Number: TJ-C20111214). Pediatric patients were considered those aged less than or equal to 18 years. Written informed consent was obtained from all patients and parents of the children included.

### SPECT imaging of salivary gland (SSG)

SSG was performed with a dual-head Discovery NM/CT 670 SPECT/CT instrument (General Electric Medical Systems, Waukesha, WI, USA) fitted with a low-energy, general-purpose, parallel-hole collimator at standard peak energy settings (20% at 140 keV). The subject was supine, and the probe was positioned for an anterior head-and-neck projection. Dynamic images were immediately acquired in a 128×128 pixel matrix at 1 min per frame for 30 min after a bolus intravenous injection of 370MBq of ^99m^Tc-sodium pertechnetate. At 15 min post injection, each subject was administered 300 mg VitC sublingually without moving, while imaging was continued.

**Fig 1:**
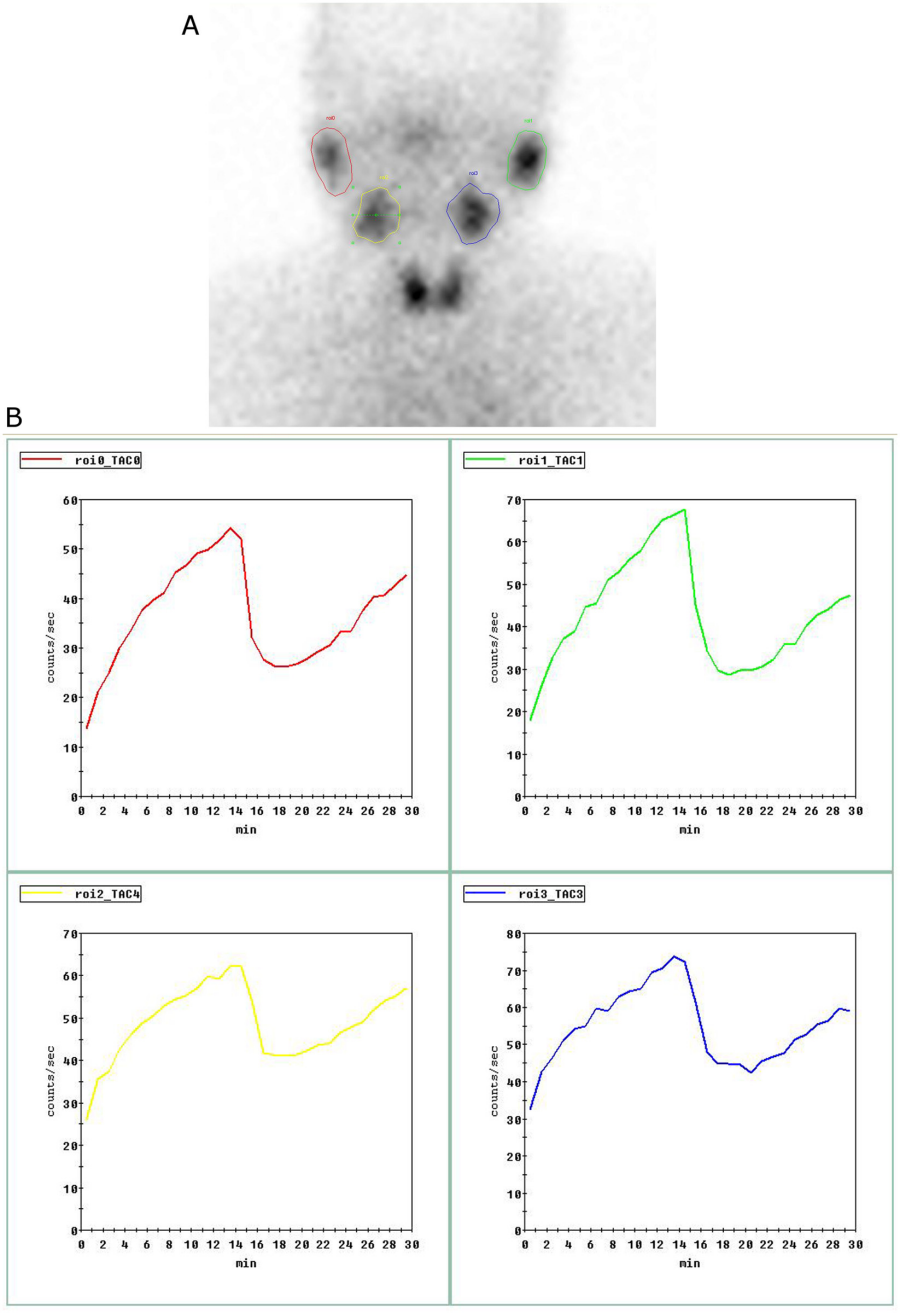
Regions of interest (ROIs) in SPECT imaging of salivary gland (SSG). The images of bilateral parotid and submandibular glands at 14min postinjection of radiotracer in the left panel (a). The time-activity curves for right parotid gland (red), left parotid gland (green), right submandibular gland (yellow) and left submandibular gland (blue) in the right panel (b).

The images were interpreted by three nuclear medicine physicians. Regions of interest (ROIs) were drawn on the dynamic images of parotid and submandibular glands, and time-activity curves were generated for each gland (Fig 1). The following quantitative parameters were derived from SSG for each gland: T_max,_ the time needed to achieve the maximum counts; UI_max_, (C_p_-C_0_)/C_0_ (C_p_, the maximum counts before VitC stimulation; C_0_ the counts at 1 min); T_s_, the time needed to achieve the minimum counts after VitC stimulation; and EF_s_, (C_p_-C_v_)/(C_p_-C_b_) (C_v_, the minimum counts after VitC stimulation; C_b_, background count which is obtained from the average count of bilateral frontal regions)

### Minor salivary gland biopsy

A written consent was obtained from each patient before minor salivary gland biopsy. The labial salivary glands were routinely fixed, sectioned, and stained with hematoxylin and eosin. Then the specimens were examined by two experienced pathologists for lymphocytic infiltration and the number of lymphocytic foci every 4mm^2^ tissue.

### New point-scoring diagnostic system for SS based on the quantified SSG

New point-scoring diagnostic system for SS was constructed (Table 1). The point score reflects specificity of each item.

**Table 1:** The new point-scoring diagnostic system for Sjögren's syndrome: domains, categories and point scores.

Domain	Category	score
**Serology**	Negative anti-SSA/Ro and anti-SSB/La	0
	Positive anti-SSA/Ro and/or anti-SSB/La	5
**Minor salivary glands biopsy**	Normal	0
	FS ≥ 1 focus/4 mm^2^	4
**SPECT imaging of salivary gland**	EF_S_ ≥ 40%	0
	40% < EF_S_ ≥ 30%	1
	30% < EF_S_ ≥ 20%	2
	EF_S_ < 20%	3
**Ocular examination**	Normal or CFS < 3 and/or Schirmer’s I test > 5 mm/5min and/or BUT > 5s	0
	CFS ≥ 3 or Schirmer’s I test ≤ 5 mm/5min or BUT ≤ 5s	1

CFS cornea fluorescein staining, BUT breakup time, FS focus score.

All patients were rated according to the new point-scoring diagnostic system above. At the same time, 2002 AECG criteria and 2012 ACR criteria were applied to each study participant. Table 2 presents the rules for classification used in the new point-scoring diagnostic system, 2002 AECG criteria and 2012 ACR criteria. The clinical diagnosis of SS was the unified standard, and we estimated the sensitivity, specificity, positive predictive value (PPV), negative predictive value (NPV) and accuracy for each criteria set.

**Table 2:** Criteria for Sjögren's syndrome.

	New criteria*	AECG criteria**	ACR criteria***
**I. Ocular dryness symptoms**	N/A	Yes	N/A
**II. Oral dryness symptoms**	N/A	Yes	N/A
**III. Ocular examinations**	0/1	CFS ≥ 4 in van Bijsterveld’s scale or Schirmer’s I test ≤ 5 mm/5min	Keratoconjunctivitis sicca with OSS ≥ 3
**IV. Histopathology**	0/4	FS ≥ 1 focus/4 mm^2^	FS ≥ 1 focus/4 mm^2^
**V. Salivary gland involvement**	0~3	Delayed uptake, reduced concentration and/or delayed excretion of tracer	/
**VI. Serology**	0/5	Positive anti-SSA/Ro and/or anti-SSB/La	Positive anti-SSA/Ro and/or anti-SSB/La, or positive RF and ANA ≥ 1:320

CFS cornea fluorescein staining, OSS ocular staining score, RF rheumatoid factor, ANA antinuclear antibody, FS focus score, N/A not application.

Rules for classification:

* Diagnostic score 1~13.

** Presence of any four of the six domains with at least IV or VI, or presence of any three of the four domains (III, IV, V and VI).

*** Presence of any two of the three domains (III, IV and VI).

### Statistical analysis

The diagnostic scores of all patients were analyzed by Receive Operating Characteristic (ROC) curve. The area under the ROC curve (AUC) and its 95% confidence intervals provide a measure of the overall discriminative ability of the new point-scoring diagnostic system for SS. The performance among the new point-scoring diagnostic system, 2002 AECG criteria and 2012 ACR criteria was assessed using a χ^2^ test. P<0.05 was considered statistically significant. Statistical analyses were performed using SPSS v.19.0 (SPSS Inc., Chicago, IL, USA).

## Results

All patients had the screening questionnaire for oral/ocular symptoms. 98.12% (209/213) patients had serological tests (anti-SSA/B antibodies, ANA and RF), 58.69% (125/213) patients had minor salivary glands biopsies, and 59.62% (127/213) patients had ocular examinations (ocular staining score or Schirmer’s I test). It was surprising that all patients had performed SSG. Clinical diagnosis of SS was made in 149 patients ((139 women, 10 men; age range, 13–67 y; mean age, 42.36 y). There were 64 nSS patients (53 women, 11men; age range, 16–84 y; mean age, 55.00 y) in Group B.

### Quantitative parameters derived from SSG

Among all quantitative parameters, the EF_s_ values for bilateral parotid and submandibular glands in both group A (SS) were significantly lower than those for the corresponding glands in group B (nSS) (Table 3).

**Table 3:** Comparison of quantitative parameters from SPECT imaging of salivary gland between group A and group B.

Quantitative parameters	Group A (SS)	Group B (nSS)	t value	*P*
**LPG**				
**T**_**max**_ **(min)**	14.17±1.21	14.39±2.09	0.77	0.44
**UI**_**max**_	0.97±0.77	1.14±0.49	1.57	0.12
**T**_**s**_ **(min)**	3.53±1.39	3.14±1.07	1.99	0.06
**EF**_**s**_	0.30±0.39*	0.51±0.24	4.96	0.00
**RPG**				
**T**_**max**_ **(min)**	14.30±1.65	14.16±1.45	0.59	0.56
**UI**_**max**_	1.00±0.88	1.04±0.42	0.49	0.63
**T**_**s**_ **(min)**	3.37±1.32	3.06±1.04	1.65	0.10
**EF**_**s**_	0.34±0.24*	0.49±0.24	4.09	0.00
**LSG**				
**T**_**max**_ **(min)**	13.92±1.68	13.67±2.07	0.92	0.36
**UI**_**max**_	0.35±0.48	0.45±0.26	1.48	0.14
**T**_**s**_ **(min)**	3.79±1.27	3.88±1.75	0.55	0.59
**EF**_**s**_	0.17±0.18*	0.33±0.19	6.09	0.00
**RSG**				
**T**_**max**_ **(min)**	14.02±1.47	13.78±2.00	0.97	0.33
**UI**_**max**_	0.35±0.48	0.41±0.27	0.99	0.06
**T**_**s**_ **(min)**	3.87±1.35	3.56±1.54	1.44	0.15
**EF**_**s**_	0.17±0.20*	0.37±0.22	6.42	0.00

* P<0.05, compared with group B (nSS).

LPG left parotid gland, RPG right parotid gland, LSG left submandibular gland, RSG right submandibular gland.

### Minor salivary gland biopsy

125 patients with minor salivary gland biopsy included 97 SS patients and 28 nSS patients. A positive result for minor salivary gland biopsy was defined as showing lymphocytic infiltration with focus score ≥ 1. The sensitivity and specificity for minor salivary gland biopsy in differentiate SS patients from nSS patients were 60.63% and 82.14%, respectively.

### Performance evaluation of the new point-scoring diagnostic system for SS

**Fig 2:**
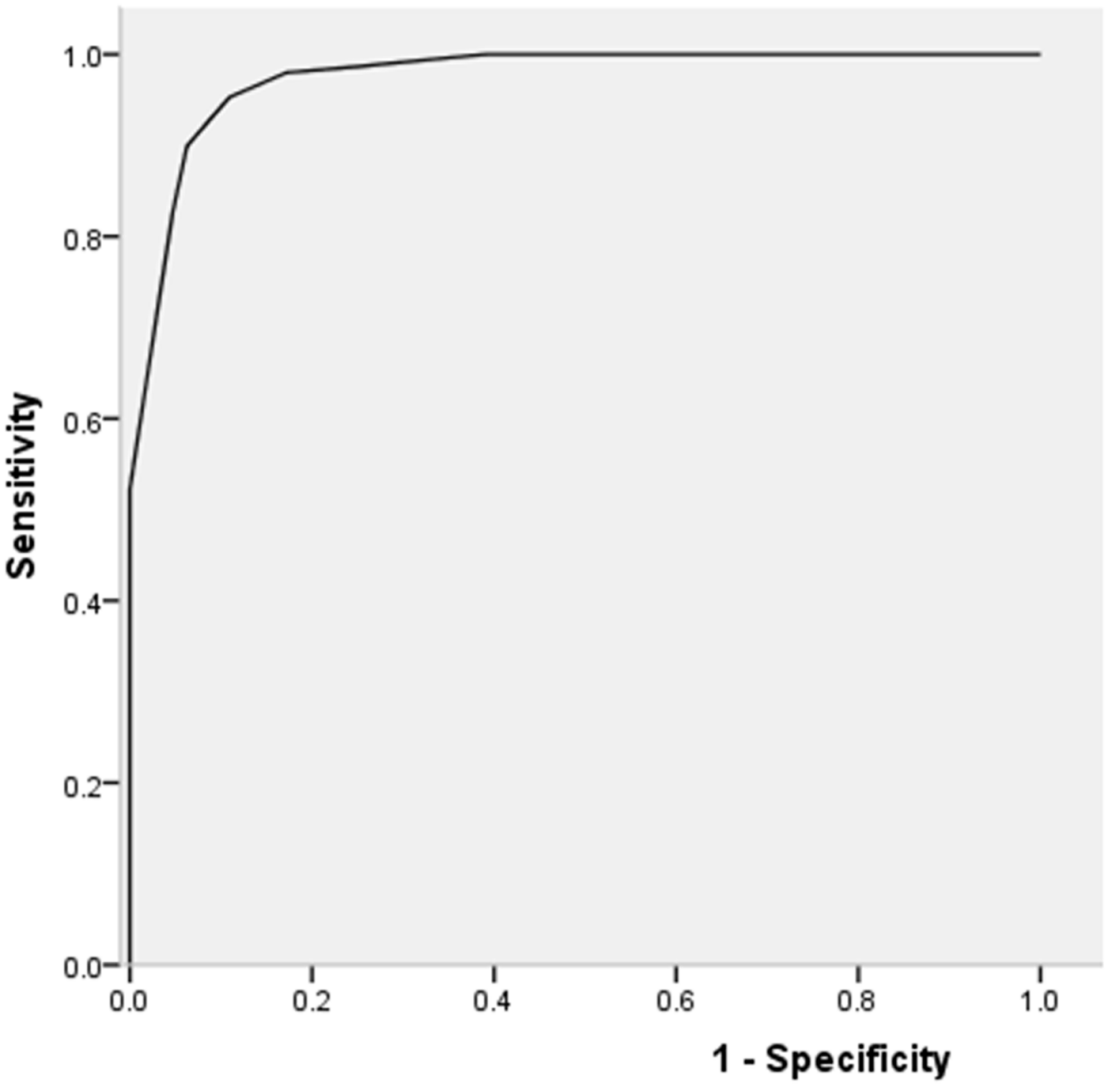
Receiver operating characteristic curve of the new diagnostic system for Sjögren's syndrome (SS). The curve plots the relationship between sensitivity and 1-specificity for different cutoff levels. When 7.0 for the diagnostic scores of suspected SS patients was used as the cut-off point, the maximum value of Youden’s index was achieved as 0.836.

The ROC area of the new point-scoring diagnostic system for SS was 0.975, and its 95% confidence intervals were 0.955 to 0.995. When 7.0 was used as the cut-off points, the sensitivity, specificity, PPV, NPV and accuracy in diagnosing SS were 89.93% (134/149), 93.75% (60/64), 97.10% (134/138), 80.00% (60/75) and 91.08% (194/213), respectively (Fig 2 and Table 4).

**Table 4:** The performance evaluation of classification criteria in 213 patients with suspicious SS.

	Clinical criteria	New criteria diagnostic score		AECG criteria		ACR criteria	
		≥ 7	< 7	Positive	Negative	Positive	Negative
**SS**	14964	134	15	80	69	88	61
**nSS**	64	4	60	5	59	3	61
**Sensitivity**	/	89.93%*		53.69%^##^		59.06%	
**Specificity**	/	93.75%^#^		92.19%^##^		95.31%	

*χ^2^ = 52.02, P = 0.00, compared among the new criteria, the 2002 AECG criteria and the 2012 ACR criteria; χ^2^ = 48.34, P = 0.00, compared with the 2002 AECG criteria; χ^2^ = 37.37, P = 0.00, compared with the 2012 ACR criteria.

^#^χ^2^ = 0.53, P = 0.77, compared among the new criteria, the 2002 AECG criteria and the 2012 ACR criteria; χ^2^ = 0.12, P = 0.73 compared with the 2002 AECG criteria; χ^2^ = 0.15, P = 0.70, compared with the 2012 ACR criteria.

^##^Compared between the 2002 AECG criteria and the 2012 ACR criteria, the sensitivity (χ^2^ = 0.87, P = 0.35) and specificity (χ^2^ = 0.53, P = 0.47) didn’t show significantly different.

And taking the clinical diagnosis as the unified standard, the sensitivity, specificity, PPV, NPV and accuracy for 2002 AECG criteria and 2012 ACR criteria to classify SS patients were 53.69% and 59.06% (139/213 and 149/213), 92.19% and 95.31% (69/74 and 61/64), 94.12% and 96.70% (80/85 and 88/91), 53.91% and 50.00% (69/128 and 61/122), 65.26% and 69.95% (139/213 and 149/213), respectively (Table 4). The above data indicate that the new point-scoring diagnostic system for SS based on the quantified SSG keeps the specificity comparatively to 2002 AECG criteria and 2012 ACR criteria, but improves sensitivity significantly.

**Fig 3:**
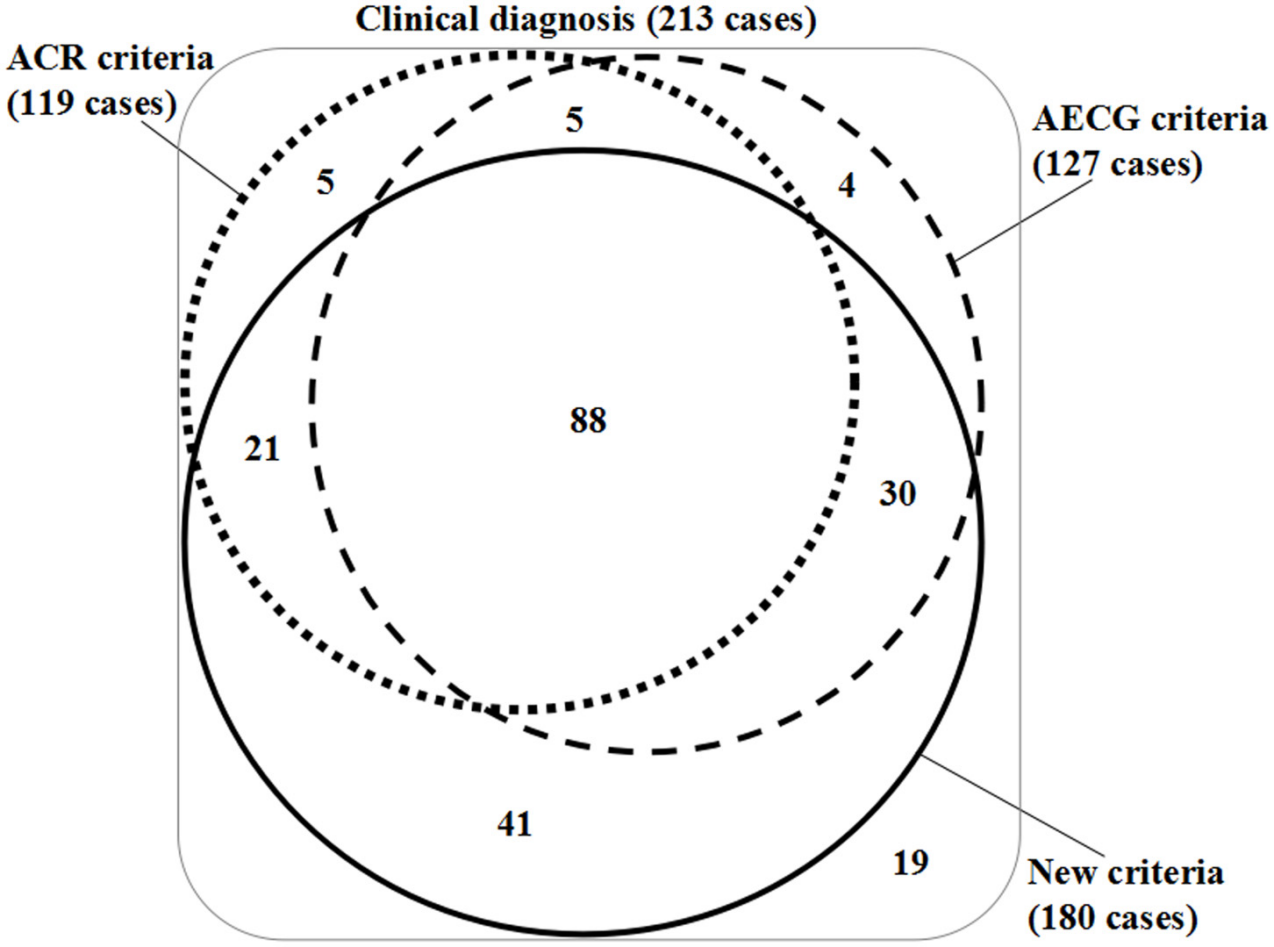
The number of patients with definitive diagnostic results consistent with the clinical diagnostic results. Area-proportional Venn diagrams visualizing interrelationships among the new point-scoring diagnostic system, the criteria proposed by the American-European Consensus Group (AECG) and the criteria proposed by the American College of Rheumatology (ACR).

Furthermore, it is very common that the patients didn’t have all the data points because of many reasons such as refusal, economic reason and so on, so that they couldn’t meet the criteria in the clinical settings. In this study, the new point-scoring diagnostic system for SS were inconclusive in 10.80% (23/213) of patients, 2002 AECG criteria were inconclusive in 22.07% (47/213) of patients and 2012 ACR criteria were inconclusive in 31.92% (68/213) of patients because of lacking the related data. To compare these three criteria equally, after excluding these patients, the sensitivity, specificity, PPV, NPV and accuracy for the new point-scoring diagnostic system, 2002 AECG criteria and 2012 ACR criteria to classify SS patients were 95.71% (134/140), 70.18% (80/114) and 79.28% (88/111); 92.00% (46/50), 90.38% (47/52) and 91.18% (31/34); 97.10% (134/138), 94.12% (80/85) and 96.70% (88/91); 88.46% (46/52), 58.02% (47/81) and 57.41% (31/54); 94.74% (180/190), 76.51% (127/166) and 82.07% (119/145), respectively (Table 5 and Fig 3), which indicates that current new point-scoring diagnostic system may be superior to 2002 AECG criteria and 2012 ACR criteria with higher sensitivity and similar specificity in the diagnosis of SS. In addition, it also has good feasibility in the clinical settings.

**Table 5:** The performance evaluation of the three criteria in Sjögren's syndrome after excluding the patients lacking of the related data.

	New criteria diagnostic score (n = 190)		AECG criteria (n = 166)		ACR criteria (n = 145)	
	≥ 7	< 7	Positive	Negative	Positive	Negative
**SS**	134	6	80	34	88	23
**nSS**	4	46	5	47	3	31
**Sensitivity**	95.71%*		70.18%^##^		79.28%	
**Specificity**	92.00%^#^		90.38%^##^		91.18%	

*χ^2^ = 30.04, P = 0.00, compared among the new criteria, the 2002 AECG criteria and the 2012 ACR criteria; χ^2^ = 30.89, P = 0.00, compared with the 2002 AECG criteria; χ^2^ = 16.37, P = 0.00, compared with the 2012 ACR criteria.

^#^χ^2^ = 0.08, P = 0.96, compared among the new criteria, the 2002 AECG criteria and the 2012 ACR criteria; χ^2^ = 0.08, P = 0.77 compared with the 2002 AECG criteria; χ^2^ = 0.02, P = 0.89, compared with the 2012 ACR criteria.

^##^Compared between the 2002 AECG criteria and the 2012 ACR criteria, the sensitivity (χ^2^ = 0.46, P = 0.12) and specificity (χ^2^ = 0.02, P = 0.90) didn’t show significantly different.

## Discussion

There have been more than 10 classification or diagnostic criteria published for SS since 1965 [4,5,6,7,15,16]. Among them, 2002 AECG criteria and 2012 ACR criteria have better specificity than any others, as they require evidence of autoimmunity from serologic tests and salivary gland biopsy [4,10,11]. In this study, the high specificity was found in 2002 AECG criteria (92.19%) and 2012 ACR criteria (95.31%) to classify SS patients, however, the unsatisfactory sensitivity was also showed for 2002 AECG criteria (53.69%) and 2012 ACR criteria (59.06%). Similar results can be in other studies [2,10,11,17]. The new point-scoring diagnostic system was able to significantly improve the sensitivity for the diagnosis of SS while maintain a high specificity (Table 4).

Taking the clinical diagnosis as the unified standard for diagnosing SS, the accuracy of the new point-scoring diagnostic system, 2002 AECG criteria and 2012 ACR criteria were 91.08%, 65.26% and 69.95%, respectively. It indicated that the new point-scoring diagnostic system based on quantified SSG may have more superior performance in the diagnosis of SS compared with 2002 AECG criteria and 2012 ACR criteria. According to this study, 40.38% (86/213) and 44.13% (94/213) of patients with a clinical diagnosis of SS were missed by 2002 AECG criteria or 2012 ACR criteria, respectively, and even if both criteria are applied in parallel, 28.17% (60/213) of SS patients were still missed (Fig 3). And there were only 15.49% (33/213) of patients with a clinical diagnosis of SS missed by the new point-scoring diagnostic system. Because of including the useful substitute items, the new diagnostic system for SS could be more widely available in a variety of settings than 2002 AECG criteria and 2012 ACR criteria.

In the 2012 ACR criteria all functional or morphological tests for the salivary glands are not included, and the estimation of salivary glands only are limited to minor salivary glands biopsy. However, the pathological features of minor salivary glands is not at all the same as those of major salivary glands in SS patients, which are partially overlapped with those of chronic inflammation of minor salivary glands [8,11,18]. Furthermore, minor salivary glands biopsy may be perceived as invasive, and some patients with suspicious SS may refuse it. In this study, 41.31% patients with suspicious SS had not performed minor salivary glands biopsy. A qualitative SSG was mentioned in the 2002 AECG criteria, but it has been complained of low specificity and radiation exposure although it has high sensitivity [2,4,11,17]. In fact, radionuclide imaging of salivary gland yield only relatively low effective radiation doses compared with computed tomography (CT) for sialography [19,20,21,22], and has been increasingly accepted by clinicians and patients for it is noninvasive, convenient, and reasonably priced. To our surprise, all patients in this study were willing to perform SSG. Additionally, in order to improve the specificity of SSG, we chose T_max_, UI_max_, T_s_ and EF_s_ as assessment parameters. The EF_s_ values for bilateral parotid and submandibular glands showed significant difference between the SS patients and the nSS patients. Deceased EFs is thought to be due to a progressive reduction in exocrine function of the major salivary glands, and may be a useful quantitative parameter for differential diagnosis of SS. According to the decreasing value, quantified SSG gives 0–3 points in the new diagnostic system for SS (Table 1).

Except for quantified SSG, the new diagnostic system included the evaluation of anti-SSA/Ro antibody and anti-SSB/La antibody [8,15], minor salivary glands biopsy [18,23] and ocular examination according to the clinical features of SS (Table 1). Unlike 2012 ACR criteria, positive RF plus antinuclear antibody (ANA) titer ≥1:320 are not used as an alternative test in the absence of anti-SSA/Ro and/or anti-SSB/La because we found that they are not diagnostically equivalent in the clinical setting. And unlike 2002 AECG criteria, the subjective symptom of dry mouth and/or dry eyes is excluded because of its significant individual differences [10,24]. In addition, being not specific for SS, Schirmer’s I test and OSS [4,8] cannot make a major contribution to the diagnosis, and either positive result scores 1 point in the new diagnostic system.

Besides, many patients with suspicious SS go untested and do not receive a confirmed diagnosis. The main reasons include that the large number of tests required to fulfill the criteria for the diagnosis of SS, and that some patients do not want to perform invasive tests. The new point-scoring diagnostic system removed the limitation on the number of test items. Our results showed that less patients were excluded by the new diagnostic system (10.80%) than 2002 AECG criteria (22.07%) and 2012 ACR criteria (31.92%) for lacking the related data. It also suggested that the new point-scoring diagnostic system for SS had good feasibility in clinical settings.

Recent studies [25,26] showed that salivary gland ultrasonography was valuable for assessing major salivary gland involvement in SS, but further research work still need to be done. Other methods, such as magnetic resonance imaging (MRI) and computed tomography (CT) of salivary glands, may have limited availability, and their capability in the early stages of SS may also be questionable [27,28]. So all of the above three imaging examinations are not considered to be included in the new diagnostic system for SS.

Based on the results of this study, although the new diagnostic system could more correctly differentiate SS patients from the suspicious patients than 2002 AECG criteria and 2012 ACR criteria, a subset of patients with SS is still missed. Some new promising procedures may need to be explored, and other alternative tests may need to be developed. In addition, for achieving the widest consensus in clinical practice, a large-scale study is necessary to verify the superior accuracy of the new diagnostic system for SS.

## Conclusion

The new point-scoring diagnostic system for SS based on quantified SSG may be superior to 2002 AECG criteria and 2012 ACR criteria, with higher sensitivity and similar specificity in the diagnosis of SS. Additionally, it also has good feasibility in the clinical settings.
